# *In Vitro* Bioaccessibility of Phenolic Acids from a Commercial Aleurone-Enriched Bread Compared to a Whole Grain Bread

**DOI:** 10.3390/nu8010042

**Published:** 2016-01-13

**Authors:** Margherita Dall’Asta, Letizia Bresciani, Luca Calani, Marta Cossu, Daniela Martini, Camilla Melegari, Daniele Del Rio, Nicoletta Pellegrini, Furio Brighenti, Francesca Scazzina

**Affiliations:** 1 LS9 Interlab Group, The Laboratory of Phytochemicals in Physiology, Department of Food Science, Medical School, University of Parma, 43125 Parma, Italy; margherita.dallasta@unipr.it (M.D.); letizia.bresciani@studenti.unipr.it (L.B.); daniela.martini2@libero.it (D.M.); daniele.delrio@unipr.it (D.D.R.); 2 Department of Food Science, Medical School, University of Parma, 43125 Parma, Italy; luca.calani@unipr.it (L.C.); marta.cossu@studenti.unipr.it (M.C.); nicoletta.pellegrini@unipr.it (N.P.); furio.brighenti@unipr.it (F.B.); 3 Barilla G. e R. F.lli, Via Mantova, 166, 43122 Parma, Italy; Camilla.Melegari@barilla.com; 4 The Need for Nutrition Education/Innovation Programme (NNEdPro), University of Cambridge, Elsie Widdowson Laboratory 120 Fulbourn Road, Peterhouse Technology Park, Cambridge CB1 9NL, UK

**Keywords:** ferulic acid, whole grain bread, aleurone, bioaccessibility

## Abstract

Wheat aleurone, due to its potentially higher bioaccessibility and bioavailability of micronutrients and phenolic acids, could represent a useful ingredient in the production of commonly consumed cereal-based food. The aim of the present study was to investigate the *in vitro* bioaccessibility of phenolic acids both from an aleurone-enriched bread and from a whole grain bread. The two bread samples were firstly characterized for the phenolic acid content. An *in vitro* digestion was then performed in order to evaluate the release of phenolic acids. The results obtained suggest that the bioaccessibility of the phenolic acids in the aleurone-enriched bread is higher than in the whole grain bread. These *in vitro* results suggest the potential use of aleurone in the production of foods, and this may represent an attractive possibility to vehicle nutritionally interesting components to consumers.

## 1. Introduction

Mounting evidence indicates that diets rich in whole grain products contribute to a reduced risk of developing chronic degenerative diseases, such as cardiovascular disease, type 2 diabetes, and some cancers, and they may also play a role in body weight management and digestive health [1,2,3,4,5,6,7]. The worldwide dietary guidelines recommend replacing refined products with an increased whole grain cereal intake [5]. Despite the growing awareness about the health effects associated with the consumption of whole grain products, the consumption of whole grain products is much lower than the recommended intake in many Western countries [8].

Consumer concerns about sensory quality represents the main cause of the poor intake of whole grain foods, making efforts aimed at overcoming this perception of paramount importance [9]. New technologies allow for the isolation and reincorporation of different specific fractions of the cereal kernel in food recipes, with the objective of producing foods with the nutritional characteristics of whole grain, but with the appearance and taste of refined grain products [10]. This could represent a major step to encourage and increase the consumption of whole grain products. To this end, wheat aleurone has been proposed as a nutritionally-rich fraction for cereal-based food production [11].

Wheat aleurone is a part of the kernel, composed of a single layer of cells. It constitutes 6%–9% of the dry mass of the grain and is anatomically located between the starchy endosperm and the outer husks [12]. The aleurone comprises a layer of cells covering the surface of the endosperm and its biological function includes the accumulation and mobilization of nutrients during development and germination of the seed [13]. Although the aleurone layer is botanically considered to be a part of the endosperm, it is usually removed together with the outer fractions of the wheat during the milling process, being strongly attached to the hyaline layer, and it is usually recovered in the bran. Wheat aleurone is an important source of fiber and, due to its biological role of nutrient stock for the kernel, it is also particularly rich in high quality protein, lipids (such as plant sterols), B vitamins, minerals, and phytochemical compounds such as phenolic acids [11]. The main phenolic acids in wheat aleurone are the hydroxycinnamic acids [11], including ferulic acid, which has been described being the most abundant and, possibly, one of the major contributors to the beneficial effects associated to whole grain consumption [14]. Ferulic acid occurs mainly as dimers (diferulates) covalently bound to arabinoxylans [15], and it has an important role in the structural properties of aleurone fiber fraction. However, it is this structural characteristic that may affect the bioavailability of ferulic acid and other phenolic compounds in this matrix [16]. Recently, an increased *in vitro* bioaccessibility of phenolic acids and vitamins from two isolated aleurone fractions, when compared with the unfractionated bran conventionally used in the production of wholemeal products, has been described by our group [17]. The bioavailability of hydroxycinnamates from the same isolated wheat aleurone fractions was also investigated *in vivo*, confirming an increased 24-h excretion of phenolic metabolites/catabolites, mainly ferulic acid, in rats fed with wheat aleurone-rich pellets compared to the regular control diet [18]. Consequently, aleurone could represent a beneficial ingredient in production of cereal-based food. 

The aim of the present work was to investigate the *in vitro* bioaccessibility of phenolic acids, both from an aleurone-enriched bread and from a whole grain bread.

## 2. Experimental Section

### 2.1. Bread Samples

Both the test commercial aleurone-enriched sliced bread (“Pan Bauletto Fior di Fibra”) and whole grain sliced bread (“Pan Bauletto Integrale”) were provided by Barilla S.p.A. The test bread is industrially produced by enriching common wheat (*T. aestivum*) flour with an aleurone-rich fraction obtained from durum wheat (*T. turgidum* var. durum). The aleurone fraction was previously described by Zaupa *et al.* [17] as aleurone A or inner part of aleurone (IN), and was subsequently used for the rats feeding in the work of Calani *et al.* [18] as aleurone A (WA-A). The ingredients of the aleurone-enriched bread (“Pan Bauletto Fior di Fibra”) were: common wheat flour (0 type, moisture ≤ 14.50%, ash ≤ 0.65% DW (dry weight), and protein ≤ 11% DW, as regulated by Italian DPR 187/2001), water, aleurone-rich fraction (9.3%), extra virgin olive oil (3.9%), yeast, wheat gluten, salt, sugar, barley flour. The total extraction rate of the two flours used for the production of aleurone-rich bread (common wheat flour and aleurone-rich fraction) was 88%–90%. The ingredients of the commercial whole grain bread were: whole grain flour from common wheat (64.2%), water, sunflower oil (4.3%), wheat gluten, yeast, dextrose, salt, and barley flour. The extraction rate of the whole grain bread corresponded to 95%–99%. The nutrient composition of the two breads is described in Table 1.

**Table 1:** Nutrient composition of the two breads.

Mean Value (100 g)	Whole Grain Bread	Aleurone-Enriched Bread
Energy (kcal)	262	261
Energy (kJ)	1101	1101
Fats (g)	5.8	4.2
Carbohydrates (g)	37.5	43.3
Fiber (g)	8.0	6.0
Proteins (g)	10.9	9.5

### 2.2. Chemicals

Ferulic acid, *p*-coumaric acid, caffeic acid, sinapic acid, pepsin from porcine gastric mucosa, pancreatin from porcine pancreas (3 × USP), and all the chemicals used in this study were purchased from Sigma-Aldrich (Steinheim, Germany). Spectra/Por^®^ dialysis membrane tubes were from Spectrum^®^ Laboratories Inc. (Rancho Dominguez, CA, USA).

### 2.3. In Vitro Digestion

The *in vitro* digestion of the breads was performed following the method of Zaupa *et al.* [17]. Three slices of each bread were minced using a household chopper (La Moulinette, Moulinex, France) and 8 g of sample were suspended in 5 mL of preheated (37 °C) phosphate buffer (20 mM) NaCl 10 mM (pH 6.9) and 25 mL of NaCl (0.9%) with 1.5 mL human saliva for 2 min. The saliva was collected from three non-smoker adult donors after thorough tooth brushing and abstinence from food and drink (except water) for at least 1 h before the experiment. The pH was then adjusted to 2.0–2.5 using HCl 8 M. Samples were then incubated at 37 °C for 2 h after the addition of 1 mL solution of NaCl (0.9%) dissolved porcine pepsin (2500 U) to mimic the gastric phase. For the intestinal step, the pH was modified to 6.9 with 5 M NaOH and each sample transferred into 20 cm dialysis tubing strips (final volume: 50 mL; 12,000–14,000 Da molecular weight cut-off) with 100 mg of pancreatin from porcine pancreas (3 × USP), sealed with plastic clamps, and incubated at 37 °C in a Dubnoff bath (ISCO, Milan, Italy), shaken at 200 rpm for 24 h into 1000 mL sealed containers with 450 mL of phosphate buffer. After 24 h of digestion dialysis, all the retained samples (inside tube/undigested sample) were stored at −20 °C prior to analysis. All *in vitro* digestions were performed in triplicate for each bread sample.

The bioaccessibility of each phenolic compound was expressed using the following calculation:

((Amount before digestion − amount in residue after digestion)/Amount before digestion) × 100

### 2.4. Extraction of Phenolic Acids

Extraction of phenolic compounds from both fresh bread samples and fractions retained within the dialysis tube after digestion was performed following method of Zaupa *et al.* [17]. For the determination of free phenolic acids, 50 mg of sample were extracted with 6 mL of water under agitation for 20 min at room temperature, followed by centrifugation at 9200× *g* for 10 min, after which the supernatant was collected and stored at −20 °C. For the extraction of bound phenolic compounds, the residue was further hydrolyzed with 1.5 mL of 2 M sodium hydroxide at room temperature for 1 h. After alkaline hydrolysis, the pH of the mixture was adjusted to 3 by adding 1.35 mL of 3 M citric acid. The bound phenolic samples were then extracted with 6 mL of ethyl acetate. The ethyl acetate extracts were evaporated to dryness *in vacuo*, the residue dissolved in methanol and stored at −20 °C. All the extracts were filtered with 0.45 µm nylon filters prior to UHPLC-MS^n^ analysis. The extractions were performed in triplicate for each fresh bread sample. Each undigested sample originating from the triplicate digestive process was extracted in duplicate, generating six extracts for phenolic analyses.

### 2.5. LC-MS^n^ Analyses

Phenolic acids in breads and undigested samples were analyzed using an Accela UHPLC 1250 equipped with linear ion-trap-mass spectrometer (LTQ XL, Thermo Fisher Scientific Inc., San Jose, CA, USA) fitted with a heated-electrospray ionization probe (H-ESI-II; Thermo Fisher Scientific Inc.). Separation was carried out by means of a C18 BlueOrchid column (50 × 2 mm; 1.8 µm particle size; Knauer, Berlin, Germany). The phenolic acids analysis was carried out in negative ionization mode, using the following conditions. The MS worked with a capillary temperature of 275 °C, while the source heather temperature was set to 45 °C. The sheath gas flow was 40 units, while auxiliary and sweep gases were set to 5 and 2 units, respectively. The source voltage was 4 kV. The capillary voltage and tube lens were −21.00 and −57.71 V, respectively. For separation of the analytes, phase A was aqueous formic acid (0.1%, *v*:*v*) and phase B was methanol/water (98:2, *v*:*v*). The mobile phase, pumped at a flow rate of 0.2 mL/min, was a 12 min linear gradient of 7% to 50% of B. Then, phase B turned up to 80% and kept for 3 min, followed by 5 min of re-equilibration time at the starting conditions. Analyses were carried out using selected reaction monitoring (SRM); *p*-coumaric acid (*m/z* 163) was fragmented using pure helium (99.999%) with a collision-induced dissociation (CID) equal to 30, generating the corresponding fragment ion at *m/z* 119; caffeic acid (*m/z* 179) yielded the corresponding fragment ion at *m/z* 135, using a CID of 29; ferulic acid (*m/z* 193) generated three daughter ions at *m/z* 134, 149, and 178, using a CID of 28; sinapic acid (*m/z* 223) generated three fragment ions at *m/z* 164, 179, and 208, with a CID of 25. The quantification of each phenolic acid was performed through a calibration curve with the respective commercial standard. Dimeric and trimeric ferulic acids with respective [M − H]^−^ values of *m/z* 385 and 577 were analyzed in full-scan MS^2^ mode and quantified as ferulic acid equivalents because of unavailability of equivalent commercial standards. Each individual sample was analyzed in duplicate. Results were expressed as milligrams per 100 grams of sample on a dry weight basis (mg/100 g DW).

### 2.6. Statistical Analysis

Data are expressed as means and standard deviation (SD). The *t* test for independent samples (*p* < 0.05) was used to identify differences between the two breads, using SPSS Version 19.0 statistical software (SPSS Inc., Chicago, IL, USA).

## 3. Results

### 3.1. Characterization and Quantification of Phenolic Profile

The phenolic profile of the two breads, including mass characteristics of each identified compound, is reported in Table 2. Caffeic, *p*-coumaric, ferulic, sinapic acid, and dimers and trimers of ferulic acid were identified as the most representative phenolics in the samples. The identification was based on the mass-to-charge ratio (*m/z*) of the molecular ion and on characteristic fragment ions. Comparison with authentic standards was performed whenever possible. The quantification of phenolic acids detected in each bread is reported in Table 3. The identified phenolic acids are mainly present as bound to the fiber both in aleurone-enriched and whole grain breads. Monomeric ferulic acid and its oligomers represent the main contributors to the total phenolic content in the two matrices, ranging from 144.8 mg/100 g DW (dry weight) for whole grain bread to 70.7 mg/100 g DW for the aleurone-enriched bread, also in accordance with the fiber content, which is higher in whole grain bread with respect to the test bread (Table 1). As expected, among the free phenolics, ferulic acid was the main identified compound, with a concentration of 0.71 and 0.41 mg/100 g DW in whole grain bread and aleurone-enriched bread, respectively, while bound ferulic acid mostly occurred as dimers in both bread samples (Table 3). Among the other detected and quantified phenolics, sinapic, *p*-coumaric acid, and caffeic acids were also present in relevant concentrations (5.43 and 5.11 mg/100 g DW in total for whole grain and aleurone-enriched breads, respectively).

**Table 2:** Mass spectral characteristics of phenolic compounds identified in bread samples.

Compound	RT (min)	[M − H]^−^ (*m/z*)	MS^2^ Ions (*m/z)*
*p*-Coumaric acid	9.18	163	119
Caffeic acid	7.38	179	135
Ferulic acid	10.06	193	149, 178, 134
Sinapic acid	10.43	223	208, 179, 164
Dimeric ferulic acid	10.21	385	341, 249, 317
Dimeric ferulic acid	11.56	385	341, 297, 317, 249
Dimeric ferulic acid	13.70	385	341, 317, 249, 297
Dimeric ferulic acid	14.27	385	341, 317, 249
Trimeric ferulic acid	13.65	577	441, 509, 533, 489, 341
Trimeric ferulic acid	15.42	577	533, 441, 509

**Table 3:** Phenolic acids in bread samples. Data are expressed both as content (mg/100 g DW, mean ± SD of 6 replicates) and as percentage contribution of individual phenolic acids to the total content.

	Whole Grain Bread		Aleurone-Enriched Bread		
**Free Compounds**	**mg/100 g DW**	**%**	**mg/100 g DW**	**%**	****
*p*-Coumaric acid	0.02 ± 0.02	2.6	0.05 ± 0.01	8.3	*
Caffeic acid	0.04 ± 0.02	5.2	0.02 ± 0.01	3.3	*
Sinapic acid	nd		0.12 ± 0.02	20.0	
Ferulic acid	0.71 ± 0.16	92.2	0.41 ± 0.04	68.3	*
Dimeric ferulic acid	nd		nd		
Dimeric ferulic acid	nd		nd		
Dimeric ferulic acid	nd		nd		
Dimeric ferulic acid	nd		nd		
Trimeric ferulic acid	nd		nd		
Trimeric ferulic acid	nd		nd		
**Bound compounds**	**mg/100 g DW**	**%**	**mg/100 g DW**	**%**	****
*p*-Coumaric acid	1.49 ± 0.40	1.0	0.82 ± 0.06	1.1	*
Caffeic acid	0.79 ± 0.16	0.5	0.26 ± 0.01	0.3	*
Sinapic acid	3.08 ± 0.71	2.1	3.84 ± 0.51	5.1	*
Ferulic acid	57.88 ± 11.78	38.7	28.46 ± 1.54	37.9	*
Dimeric ferulic acid	5.33 ± 0.72	3.6	2.66 ± 0.20	3.5	*
Dimeric ferulic acid	9.04 ± 1.71	6.0	4.07 ± 0.36	5.4	*
Dimeric ferulic acid	4.73 ± 0.83	3.2	2.35 ± 0.53	3.1	*
Dimeric ferulic acid	59.16 ± 15.25	39.6	29.64 ± 3.42	39.4	*
Trimeric ferulic acid	1.25 ± 0.69	0.8	nd		
Trimeric ferulic acid	6.67 ± 1.62	4.5	3.08 ± 0.73	4.1	*
**Total free + bound**	**mg/100 g DW**	**%**	**mg/100 g DW**	**%**	****
*p*-Coumaric acid	1.51 ± 0.42	1.0	0.87 ± 0.07	1.1	*
Caffeic acid	0.83 ± 0.18	0.6	0.28 ± 0.02	0.4	*
Sinapic acid	3.08 ± 0.71	2.1	3.96 ± 0.52	5.2	*
Ferulic acid	58.59 ± 11.90	39.0	28.87 ± 1.55	38.1	*
Dimeric ferulic acid	78.27 ± 17.54	52.1	38.72 ± 3.53	51.1	*
Trimeric ferulic acid	7.92 ± 2.06	5.3	3.08 ± 0.73	4.1	*
**Total (ferulic acid + dimeric ferulic acids + trimeric ferulic acids)**	**mg/100 g DW**	****	**mg/100 g DW**	****	****
Total ferulic acid	144.78 ± 31.04		70.67 ± 4.71		*

* Significant differences between the two breads (*p* < 0.05); nd, not detected.

### 3.2. Bioaccessibility of Phenolic Acids in Bread Samples

**Figure 1:**
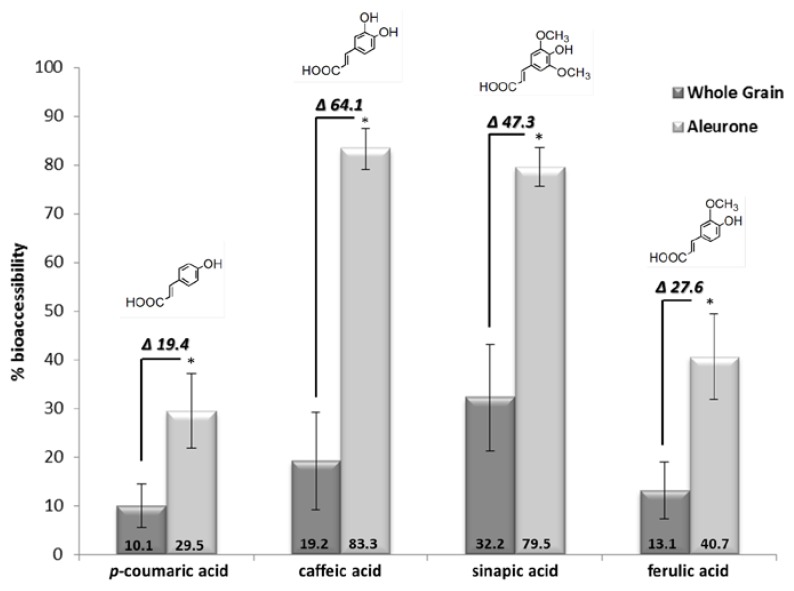
Bioaccessibility (%) of the detected phenolic acids in the two breads (mean ± SD, *n* = 12). * Significant differences between the two breads (*p* < 0.05). Δ, difference of bioaccessibility between the two breads.

Figure 1 illustrates the bioaccessibility of phenolic compounds after *in vitro* digestion. The results describe the percentage of phenolic acids released through the dialysis membrane during *in vitro* digestion with respect to the total phenolic content of the samples. The bioaccessibility of the four phenolic acids (*p*-coumaric acid, caffeic acid, ferulic acid monomers, dimers, trimers, and sinapic acid) was significantly higher from aleurone-rich bread than the whole grain bread (Figure 1). Among the phenolic acids, caffeic and sinapic acids appeared as the most bioaccessible both in aleurone-rich and whole grain breads, while total ferulic acid and *p*-coumaric acid were less bioaccessible.

## 4. Discussion

This study investigated the *in vitro* bioaccessibility of phenolic acids from a commercial bread produced with a selected aleurone fraction, compared with a whole grain bread. The aleurone fraction used for the production of the test bread, and named aleurone flour in previously published works [17,18], does not correspond to a specific histological separation as it was obtained through an industrial process.

As expected, the majority of phenolic compounds have been found to be bound to the fiber, with ferulic acid representing the main phenolic acid present in both commercial breads. The content of phenolic compounds is higher in whole grain bread than in aleurone-enriched bread, in accordance with fiber content (Table 1). The *in vitro* digestion of the two breads showed a significantly higher bioaccessibility of the four main phenolic acids detected in the aleurone-enriched bread with respect to the commercial whole grain bread, confirming the previously reported higher bioaccessibility of phenolics [17] and of antioxidant capacity [19] after *in vitro* digestion of durum wheat aleurone compared to the unfractionated bran that is commonly used for wholemeal products. In very good agreement with previous data, the bioaccessibility of ferulic acid from the aleurone flour was estimated to be around 30% [17], the same value obtained for the aleurone-enriched bread analyzed in the present study. This result suggests that phenolic acids maintain the high bioaccessibility they have shown in aleurone fraction even when aleurone fraction flour is incorporated into bread. In a recent bioavailability study carried out in an animal model, the same aleurone fraction used for the production of the test bread analyzed in the present study has been fed to rats. Several phenolic metabolites, derived from the hepatic and microbial metabolism of wheat phenolics, were found at relevant concentrations in 24 h-urine samples after both acute and chronic feedings, suggesting that aleurone fraction phenolics are actually bioavailable and not just bioaccessible [18]. 

In general, wholemeal products have been previously described as rich in phenolic compounds (mainly hydroxycinnamates), but these compounds have been described as scarcely bioaccessible and bioavailable [20,21]. Indeed, although the whole grain bread analyzed in this work provided higher levels of phenolics and fiber than its aleurone-enriched counterpart, the significantly higher bioaccessibility of phenolics in the latter would ideally contribute to a higher level of circulating phenolic metabolites *in vivo*. This fact, associated with the higher amount of micronutrients, such as minerals and B vitamins, as reported by Zaupa *et al.* [17], makes the aleurone-enriched product the best choice from a nutritional viewpoint. Thus, the results of the present study, adding to previous evidence obtained using the same fractions, suggest that the selection of products incorporating aleurone fractions, instead of whole grain, could represent a viable strategy to increase the intake of bioaccessible and bioavailable phenolics in the framework of a healthy diet. This might or might not be related to other beneficial effects, which have always been associated with the consumption of wholemeal cereal products, but evidence is also mounting that aleurone could be equally effective. Apart from the phenolic compounds, wheat aleurone has been studied for the presence of other bioactive compounds potentially able to promote a healthy status. For instance, an increase in plasma betaine was observed after the consumption of both minimally processed aleurone fraction and aleurone bread in two randomized, controlled, cross-over postprandial studies [22]. The increase was significantly higher both when the aleurone fraction was compared with another minimally-processed wheat bran fraction or the control and when the aleurone-rich bread with a white wheat flour bread control [22]. Moreover, a diet high in wheat aleurone followed by 79 healthy subjects in a parallel, single-blinded intervention study induced a significantly increased plasma betaine after four weeks compared to the control group, being also effective in lowering plasma total homocysteine (tHcy) and LDL cholesterol [23], together with significantly lowering plasma levels of C-reactive protein, which is an independent risk factor for cardiovascular disease [24].

## 5. Conclusions

The present work investigated the *in vitro* bioaccessibility of phenolic acids from a commercial aleurone-enriched bread compared with a whole grain bread. The results obtained indicate that the bioaccessibility of the phenolic acids detected in bread samples, with ferulic acid being the most represented, is higher in the test aleurone-enriched bread, suggesting a potential use of aleurone in the production of cereal based foods. This represents an attractive opportunity to supply nutritionally beneficial compounds to the consumers maintaining more acceptable sensory characteristics, including aspect and taste far from those typical of current wholemeal products.

Future studies in humans should be carried out in order to confirm the data obtained *in vitro*, with the final aim of evaluating the bioavailability of phenolic compounds in aleurone-enriched bread in humans. Finally, further clinical studies should be designed to investigate the effects of aleurone-enriched bread and other aleurone-based products on health and disease indicators in humans.
